# Cure of Drunkenness

**Published:** 1868-09

**Authors:** 


### CURE OF DRUNKENNESS.

No man doubts that Dyspeptia is a disease, if he does he ought to have it a year. Dyspeptia is an over stimulus of the stomach; it is made to work too much, its powers are overtaxed, are prematurely exhausted, and it works wildly and imperfectly; the result is physical sufferings and mental horrors many a time expressed by frenzied patients as "equal to the sufferings of the damned," giving rise to thoughts of profanity, murder, suicide and every human crime, and of which the victims in their better moments are utterly ashamed. Over stimulus of the nervous system by liquor drinking engenders dis-

*BALDWIN, THE CLOTHIER.

ease and wasting and death both to body and mind, quite as terrible to contemplate as those which follow an outraged stomach, and it is a great step in advance of the ignorance of of the past and in the direction of a true humanity to regard drunkenness as much a disease as dyspeptia or any other bodily malady, and thus regarding it the advanced minds in our hospitals, lunatic asylums and other establishments of beneficence and mercy are meeting with a most encouraging success in the treatment and cure of the disease of drunkenness ; foremost among these are Albert Day, under whose experienced supervision the Binghampton Asylum for Inebriates has been wisely placed and who reports that instead of that establishment having thirteen hundred millions of patients, it numbers just sixty six men, and this is the largest number ever contained within its walls at one time.

We have gathered two cures for drunkenness in the course of a life time. 1st, Lock a drunken person in a room and not allow him a morsel of any thing to eat which has not been soaked in whiskey. The other was performed by a lady whom we knew in our college days ; she was huge, weighing two hundred and fifty six pounds and bore one of the most honored names in our native State. Her husband came home one night hopelessly drunk ; it was his first offence, she had him put to bed carefully by the servants, laying him in a strong blanket, to keep him safe ; as soon as the servants left the room she sewed the invalid in this blanket with strong twine, the lower limbs were bound securely and the arms pinioned by the side, the head only being exposed for the purposes of ventilation. An indispensable article of housekeeping in those days in every well regulated family was what was in that early day called a cowhide, usually applied to the backs of negroes to keep them orderly and civil ; in extraordinary cases it was used to beat the dust out of furs, woolen clothing and the like ; it was rather on the confines of civilization where we were born and bred, and on urgent occasions both wives and husbands had a taste of the hide according to which had the upper hand ; in this case Mrs. H——n thinking that discipline in a family was useful and cleanliness essential began to beat the dust out of the blanket; this she did for about one hour with all the momentum of a two hundred and fifty-six pounder; yells and execrations did no good, the blanket was very dusty and needed a thorough basting, aud so did her husband, and by killing two birds with one stone she saved her strength and her husband too for he never got drunk again; wasn't it he who was the secretary of state of his native hunting grounds, not a great many years ago?

It is said that a mixture made up as follows, and taken in quantities equal to an ordinary dram, and as often as the desire for strong drink returns, will cure the worst case of drunkenness: Sulphate of Iron, five grains; peppermint water, eleven drachms; spirits of nutmeg, one drachm. This preparation acts as a tonic and stimulant, and so partially supplies the place of the accustomed liquor, and prevents the absolute physical and moral prostration that follows a sudden breaking off from the use of stimulating drinks. It is to be taken in quantities equal to an ordinary dram, and as often as the desire for a dram returns.

